# Do you mind if I take your blood pressure? Physical health monitoring of children and young people on ADHD medication amidst a pandemic

**DOI:** 10.1192/bjo.2021.497

**Published:** 2021-06-18

**Authors:** Emma Davies, Maham Khan, Claire Jones

**Affiliations:** Surrey and Borders NHS Foundation Trust

## Abstract

**Aims:**

To establish whether physical health monitoring for CYP on ADHD medication is according to NICE guidance (2018).

To determine the impact of COVID-19 pandemic restrictions on physical health monitoring for CYP on ADHD medication.

Attention deficit hyperactivity disorder (ADHD) is a common neurodevelopmental disorder, characterised by a persistent pattern of inattention and/or hyperactivity-impulsivity, directly impacting on academic, occupational, or social functioning. It affects between 1-5% of children and young people (CYP) most often presenting in early-mid childhood.

Pharmacological treatment can be considered in CYP if certain criteria are met, where licensed medications include methylphenidate, dexamfetamine, lisdexamfetamine, atomoxetine and guanfacine. Stimulant and non-stimulant medications require frequent physical health monitoring due to their side effects including an increase in blood pressure and/or heart rate, loss of appetite, growth restriction and tics.

**Method:**

Standards and criteria were derived from the NICE guidance (2018), whilst local trust policies were reviewed, demonstrating discrepancies. Standards were expected to be met for 100% of patients.

Electronic patient records were reviewed retrospectively from a representative cohort of CYP reviewed by clinicians in a community CAMHS service during March-November 2020. Data were entered manually into a spreadsheet for evaluation.

**Result:**

A total of 27 CYP records were reviewed, average age 13yo, on a range of stimulant/non-stimulant preparations.

5 (19%) had height checked every 6 months, with 4 delayed to 7-8 months.

For those >10yo, only 5 (19%) had weight checked every 6 months.

Only 2 (7%) had their height and weight plotted on a growth chart and reviewed by the healthcare professional responsible for treatment.

Just 4 (15%) had heart rate and blood pressure recorded before and after each dose change, whilst similarly only 4 (not the same) had these parameters recorded every 6 months.

17 patients were reviewed by telephone/video call, where 5 patients provided physical health parameters (measured at home).

**Conclusion:**

Across all parameters, standards are not being met for the required physical health monitoring for CYP on ADHD medication.

The COVID-19 pandemic has significantly changed the working conditions for community teams, impacting face to face reviews, creating challenges for physical health monitoring.

Our ongoing implementations for change include the use of a proforma for physical health measurements, improving psychoeducation for families, exploring potential barriers with senior colleagues and collaborating with pharmacy colleagues to update local guidelines in accordance with the latest NICE recommendations. We aim to re-audit in June 2021.

